# Association of Vascular Endothelial Growth Factor (VEGF) and Mouse Model Minute 2 (MDM2) Polymorphisms With Diabetic Retinopathy in a Northwest Indian Population: A Case-Control Study

**DOI:** 10.7759/cureus.62996

**Published:** 2024-06-23

**Authors:** Manroop Singh Buttar, Kamlesh Guleria, Swarkar Sharma, AJS Bhanwer, Vasudha Sambyal

**Affiliations:** 1 Department of Human Genetics, Guru Nanak Dev University, Amritsar, IND; 2 Centre for Molecular Biology, Central University of Jammu, Samba, IND; 3 Department of Genetics, Sri Guru Ram Das Institute of Medical Sciences and Research, Amritsar, IND

**Keywords:** genetic opthalmology, polymorphism, mcp-1, capn10, human genetics, diabetic retinopathy, genetics

## Abstract

Introduction: Diabetic retinopathy (DR), a microvascular complication of type 2 diabetes (T2D), results from complex interactions of genetic and environmental factors. Vascular endothelial growth factor (VEGF) and mouse model minute 2 (MDM2)are upregulated in the retina due to diabetes, which increases the risk of DR. VEGFA and MDM2 genetic variations can influence DR risk. The present case-control study was conducted to evaluate the association of VEGFA and MDM2 promoter variants with DR in a population from Punjab, Northwest India.

Methods: A total of 414 DR patients, 425 T2D patients without DR, and 402 healthy controls were screened for *VEGFA *-2578C/A (rs699947), *VEGFA *-2549I/D (rs35569394), *VEGFA *-7C/T (rs25648), and *MDM2* rs3730485 polymorphisms using polymerase chain reaction (PCR)-based methods.

Results: *VEGFA *-2549 I allele (OR = 1.35 (1.00-1.81), p = 0.043) and II genotype (OR = 1.78 (1.00-3.15), p = 0.047) were significantly associated with increased risk of DR. *VEGFA *-7 CT genotype conferred reduced risk of DR (OR = 0.28 (0.20-0.38); p = <0.001). *VEGFA *-2578 and *MDM2 *rs3730485 showed no significant association with DR. A-I-T (OR = 0.30 (0.20-0.44); p = <0.001) and C-D-T (OR = 0.33 (0.16-0.65); p = 0.002) haplotypes of rs699947-rs35569394-rs25648 polymorphisms showed decreased risk of DR.

Conclusions: I allele and II genotype of *VEGFA *-2549, CT genotype of *VEGFA *-7, and C-I-C and A-D-C haplotypes of rs699947-rs35569394-rs25648 polymorphisms were significantly associated with DR risk in a Northwest Indian population. This is the first study worldwide to report DR risk with VEGFA promoter variants together.

## Introduction

Diabetes mellitus (DM) is a group of metabolic disorders characterized by high blood glucose levels and an increased risk of developing a number of serious health problems, resulting in higher medical care costs, reduced quality of life, and increased mortality [1]. Diabetic retinopathy (DR) is one of the major microvascular complications associated with chronic hyperglycemia and the leading cause of preventable blindness worldwide [2]. Type 2 diabetes (T2D) represents more than 90% of the whole diabetic population in the world [3] and is responsible for 9% of global mortality corresponding to four million deaths per year. According to the International Diabetes Federation (IDF), there are nearly 65.1 million diabetic individuals in India, and the number is further expected to reach 109 million by the year 2035 [4]. Despite the documented increase in the prevalence of diabetes across the globe, still there is a scarcity of data on the prevalence and severity of DR [5]. There are very limited epidemiological studies emphasizing the prevalence of DR in rural and urban populations in India [6,7]. The overall prevalence of DR was reported to be around 38.3% in T2D cases in India [8]. Diabetes-induced blindness in working adults in India is expected to increase from 4.2 million in 2020 to over six million by 2030 [9]. DR begins with microvascular complications in the photoreceptor cells of the retina and is characterized by increased vascular permeability, progressive vascular occlusion, and neovascularization (NV), which results in the degeneration of retinal cells/tissues that finally affects vision [10]. NV is the process of forming new vasculature by vasculogenesis and angiogenesis [11]. The initial factors and determinants of ocular NV are hypoxia and oxidative stress in the outer retina [12]. Hyperglycemia induces hypoxia in the retina of diabetic individuals. Hypoxia stimulates the expression of factors such as vascular endothelial growth factor (VEGF) and mouse model minute 2 (MDM2) [13].

VEGF is a multifunctional cytokine that promotes angiogenesis and vascular permeability [14,15]. Under physiological conditions, VEGF is expressed at low levels in the eye [16]. Under pathological circumstances, the expression of VEGF is upregulated, and its overexpression promotes vessel endothelial cell proliferation, migration, tube formation, and sprouting, serving as a contributing factor for DR [17]. VEGF is also considered a primary initiator of proliferative DR (PDR) and a potential mediator of non-PDR (NPDR) [18]. Hence, the *VEGF* gene and its polymorphic variants may play crucial roles in DR, characterized by impaired vascular permeability and neovascularization [19]. However, the association between *VEGF *gene polymorphisms and the susceptibility to DR, PDR, and NPDR has not been completely established [15-19]. Human *VEGFA *(Gene ID: 7422) is located on 6p21.1, spans over 16 kb, and consists of nine exons (https://www.ncbi.nlm.nih.gov/gene/7422). It is reported to be highly polymorphic in the promoter region, 5′ untranslated region (UTR), and 3′ UTR [20]. There are reports on the association of these genetic variations with altered serum and urine VEGF levels [20,21]. *VEGFA* -2578 C/A (rs699947), *VEGFA* -2549 I/D (rs35569394), and *VEGFA* -7 C/T (rs25648) polymorphisms have been implicated in a number of diseases with angiogenic basis; hence, they are polymorphisms of particular interest [22,23].

The *MDM2* gene encodes the MDM2 homolog protein, which is a primary negative regulator of p53 [24]. The tumor suppressor protein p53 controls many important cellular events, including apoptosis and cell proliferation [25]. It has been reported that p53 is active in the absence of MDM2, triggering apoptosis or profound inhibition of cell proliferation [26]. Meanwhile, elevated MDM2expression causes persistent cell growth with significant DNA damage, which supports tumorigenesis due to loss of p53 control [27]. Under hypoxic conditions, MDM2 is overexpressed and activates hypoxia-inducible factor 1 (HIF1) in a p53-independent pathway, and HIF1 upregulates VEGF [28-30]. In addition, MDM2 directly interacts with and stabilizes VEGF mRNA and increases its translation, which is one of the main vasoactive gene products causing NV [31]. A previous study reported that, in the diabetic state, the deletion of MDM2 causes activation of endothelial p53, which reduces vasodilatation and angiogenesis, reducing the risk of PDR [32]. *MDM2 *rs3730485 is an insertion/deletion polymorphism of 40 bps in the promoter P1, and the deletion allele has been shown to reduce transcription [33-35].

The interplay between MDM2, p53, HIF1, and VEGF might be one of the key factors for the development of DR. The present study is designed to detect any association of *VEGFA* -2578C/A (rs699947), *VEGFA* -2549I/D (rs35569394), *VEGFA* -7C/T (rs25648), and *MDM2* rs3730485 promoter polymorphisms with the DR in a population from Punjab, Northwest India. These four polymorphisms are present in the promoter region, and other polymorphisms present in that area are mostly in linkage disequilibrium with the selected four. Thus, evaluating these four could also shine a light on the ones not included in the study. To the best of our knowledge, there has been no previous reported study worldwide on the association of *MDM2* rs3730485 polymorphism with the DR. This is the first study worldwide to investigate the association of *VEGFA* rs699947, rs35569394, and rs25648 polymorphisms together with DR.

## Materials and methods

Study subjects

In the present study, a total of 414 unrelated DR patients, 425 unrelated T2D patients without DR, and 402 unrelated age-matched healthy controls (CN) from Punjab, Northwest India, were included. The sample size was estimated using the power of study analysis, explained in the statistical analysis below. T2D was defined according to the American Diabetes Association diagnostic criteria [36]. Type 1 diabetic patients and T2D patients with other metabolic complications were excluded from the study. DR cases were diagnosed by ophthalmologists at Dr. Sohan Singh Eye Hospital, Amritsar, Punjab, India, based on a comprehensive ophthalmological examination, including fundus examination and fundus photography based on three 45° field tests per eye every year. Retinopathy was diagnosed according to the Early Treatment Diabetic Retinopathy Study (ETDRS) criteria: the presence of microaneurysms, hemorrhages, cotton wool spots, intra-retinal microvascular abnormalities, hard exudates, venous beading, and new vessels [37]. The DR patients were further categorized into 256 non-proliferative diabetic retinopathy (NPDR) patients and 158 proliferative diabetic retinopathy (PDR) patients. Controls were randomly selected on the basis of fasting blood sugar (FBS) and random blood sugar (RBS) levels with no previous history of diabetes and were ethnicity-matched with patients. Individuals with a family history of diabetes in first-degree relatives or any other systemic complications were not included in the control group. A written informed consent was obtained from all the study subjects. A 5-mL intravenous peripheral blood sample from each subject was collected in ethylenediaminetetraacetic acid (EDTA)-coated vials. The study was approved by the Institutional Ethics Committee of Guru Nanak Dev University, Amritsar, Punjab, India (Letter No. 573/HG, Dated- 29/03/2018).

Genetic analysis

**Figure 1:**
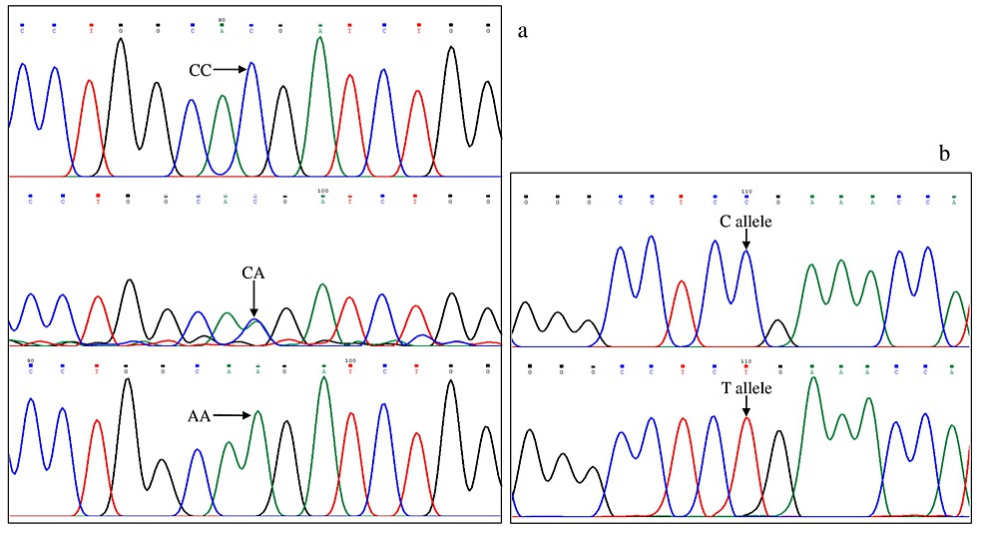
Part of electropherograms (forward strand) showing CC, CA, and AA genotypes of VEGFA rs699947 (a) polymorphism. Part of electropherograms (forward strand) showing C- and T-specific primer sequencing results of VEGFA rs25648 (b) polymorphism

**Figure 2:**
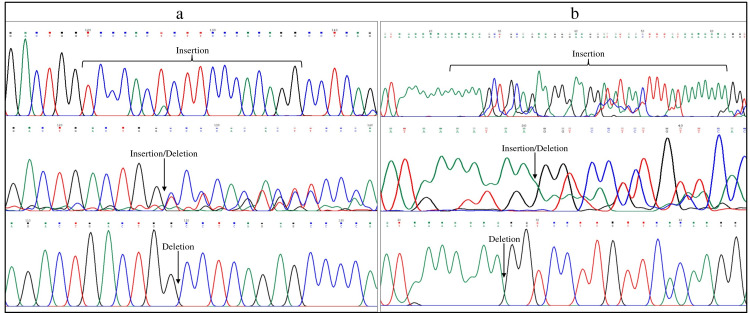
Part of electropherograms (forward strand) showing II, ID, and DD genotypes of VEGFA rs35569394 (a) and MDM2 rs3730485 (b) polymorphisms

Genomic DNA was extracted from blood using the salt precipitation method with some modifications [38]. The purity and quantity of DNA samples were checked on ethidium bromide-stained 1% agarose gel. Genotyping of *VEGFA *-2578 C/A polymorphism was performed by the PCR-RFLP method, whereas *VEGFA* -7 C/T polymorphism was genotyped using the amplification refractory mutation system (ARMS)-polymerase chain reaction (PCR). *VEGFA *-2549 I/D and *MDM2* rs3730485 I/D polymorphisms were genotyped using direct PCR. The details of primers, reaction conditions, and other details of polymorphisms are given in Table 1. The PCR results were checked using agarose gel electrophoresis. To ensure genotyping accuracy, positive and negative controls were used in every batch of reactions. The PCR results were validated by Sanger sequencing of 10% randomly selected samples (Figures 1, 2).

**Table 1:** Details of VEGFA polymorphisms and genotyping conditions used for screening

Polymorphism (RefSNP)	Location	Genotyping method	Annealing temperature	Restriction enzyme used	Allele and Fragment size (bp)	Primer reference
VEGFA−2578C/A (rs699947)	Promoter	PCR-RFLP	59°C	BglII	C- 459	[39]
					A- 247, 212	
VEGFA −2549I/D (rs35569394)	Promoter	Direct PCR	55°C	-	D- 211	[40]
					I- 229	
VEGFA −7C/T (rs25648)	5′ UTR	ARMS-PCR	60°C	-	Control- 425	[41]
					C and T- 183	
MDM2 (rs3730485)	Promoter	Direct PCR	58°C	-	I- 287	[42]
					D-247	

Statistical analysis

Data were analyzed using the Statistical Product and Service Solutions (SPSS, version 16.0; SPSS Inc., Chicago, IL). Power analysis was done using the online CaTS-GAS power calculator (https://csg.sph.umich.edu/abecasis/cats/gas_power_calculator/) with the following parameters: additive disease model, population risk of 13% for T2D, and minor allele frequency (MAF) of 46.3% for *VEGFA* -2578 C/A, 46.1% for *VEGFA* -2549 I/D, 14.3% for *VEGFA* -7 C/T, and 23% for *MDM2* rs3730485 at p=0.05. The continuous variables were represented as means ± standard deviations (SD). The Hardy-Weinberg equilibrium (HWE), allele frequencies, genotype frequencies, and genotype-genotype combinations were analyzed using the c2 test and odds ratio (OR) with 95% CI. The Haplotype analysis was done using the online SNPStats web tool (https://www.snpstats.net/start.htm) [43]. Lewontin’s standardized disequilibrium coefficient (D′) and correlation coefficient (r2) were calculated using SHEsis software [44]. The p-value for the level of significance was set to be less than 0.05 in all analyses.

## Results

Genotype and allele analysis

Genotype frequencies of all polymorphisms studied in the present study were in agreement with HWE (p > 0.05) in healthy controls. The allele distribution, genotype distribution, and genetic model analyses in DR, T2D, and controls have been given in Tables 2, 3.

**Table 2:** Distribution of genotype and allele frequencies of VEGFA and MDM2 polymorphisms in total subjects, female, male, NPDR, and PDR groups The data have been represented as n (number), %, OR: odds ratio, and p-value. p-value < 0.05 was considered significant. Significant p-values are displayed in bold. CN: Healthy Controls, DR: Diabetic Retinopathy, HWE: Hardy-Weinberg Equilibrium, MDM2: Mouse Model Minute 2, NPDR: Non-Proliferative Diabetic Retinopathy, PDR: Proliferative Diabetic Retinopathy, T2D: Type 2 Diabetes, VEGFA: Vascular Endothelial Growth Factor

Total Samples													
	DR (n= 414)			T2D (n=425)	CN (n= 402)		DR vs. CN		T2D vs. CN		DR vs. T2D		
							OR (95% CI)	p-value	OR (95% CI)	p-value	OR (95% CI)	p-value	
VEGFA -2578 C/A (rs699947)													
Genotype													
CC	125 (30.2)			116 (27.3)	124 (30.8)		Reference		Reference		Reference		
CA	199 (48.1)			213 (50.1)	184 (45.8)		1.07 (0.78-1.48)	0.666	1.24 (0.90-1.71)	0.193	0.87 (0.63-1.19)	0.379	
AA	90 (21.7)			96 (22.6)	94 (23.4)		0.95 (0.65-1.39)	0.791	1.09 (0.75-1.60)	0.652	0.87 (0.59-1.28)	0.476	
Allele													
C	449 (54.2)			445 (52.4)	432 (53.7)		Reference		Reference		Reference		
A	379 (45.8)			405 (47.6)	372 (46.3)		0.98 (0.81-1.19)	0.841	1.06 (0.87-1.28)	0.575	0.93 (0.77-1.12)	0.442	
HWE	p=0.519			p=0.925	p=0.111								
VEGFA -2549 I/D (rs35569394)													
Genotype													
DD	117 (28.3)			109 (25.7)	124 (30.8)		Reference		Reference		Reference		
ID	202 (48.8)			216 (50.8)	186 (46.3)		1.15 (0.83-1.59)	0.392	1.32 (0.96-1.83)	0.092	0.87 (0.63-1.20)	0.404	
II	95 (22.9)			100 (23.5)	92 (22.9)		1.09 (0.75-1.60)	0.644	1.24 (0.84-1.81)	0.277	0.89 (0.60-1.30)	0.532	
Allele													
D	436 (52.7)			434 (51.1)	434 (54)		Reference		Reference		Reference		
I	392 (47.3)			416 (48.9)	370 (46)		1.05 (0.87-1.28)	0.592	1.12 (0.93-1.36)	0.235	0.94 (0.77-1.14)	0.512	
HWE	p=0.663			p=0.727	p=0.168								
VEGFA -7 C/T (rs25648)													
Genotype													
CC	334 (80.7)			224 (52.7)	297 (73.9)		Reference		Reference		Reference		
CT	78 (18.8)			189 (44.5)	101 (25.1)		0.69 (0.49-0.96)	0.028	2.48 (1.84-3.34)	<0.001	0.28 (0.20-0.38)	<0.001	
TT	2 (0.5)			12 (2.8)	4 (1.0)		0.44 (0.08-2.45)	0.351	3.98 (1.27-12.5)	0.018	0.11 (0.02-0.50)	0.004	
Allele													
C	746 (90.1)			637 (74.9)	695 (86.4)		Reference		Reference		Reference		
T	82 (9.9)			213 (25.1)	109 (13.6)		0.70 (0.52-0.95)	0.022	2.13 (1.65-2.75)	<0.001	0.33 (0.25-0.43)	<0.001	
HWE	p=0.256			p=0.001	p=0.149								
MDM2 (rs3730485)													
Genotype													
CC	251 (60.6)			241 (56.7)	234 (58.2)		Reference		Reference		Reference		
CT	136 (32.9)			161 (37.9)	151 (37.6)		0.84 (0.63-1.12)	0.241	1.04 (0.78-1.38)	0.812	0.81 (0.61-1.08)	0.155	
TT	27 (6.5)			23 (5.4)	17 (4.2)		1.48 (0.79-2.79)	0.223	1.31 (0.68-2.52)	0.412	1.13 (0.63-2.02)	0.688	
Allele													
C	638 (77.1)			643 (75.6)	619 (77)		Reference		Reference		Reference		
T	190 (22.9)			207 (24.4)	185 (23)		0.99 (0.79-1.26)	0.976	1.08 (0.86-1.35)	0.521	0.93 (0.74-1.16)	0.498	
HWE	p=0.148			p=0.337	p=0.228								
Female Group													
	DR (n= 147)	T2D (n=190)			CN (n= 234)		DR vs. CN		T2D vs. CN		DR vs. T2D		
							OR (95% CI)	p-value	OR (95% CI)	p-value	OR (95% CI)	p-value	
VEGFA -2578 C/A (rs699947)													
Genotype													
CC	45 (30.6)	56 (29.5)			77 (32.9)		Reference		Reference		Reference		
CA	65 (44.2)	89 (46.8)			106 (45.3)		1.05 (0.65-1.70)	0.844	1.15 (0.74-1.80)	0.527	0.91 (0.55-1.51)	0.711	
AA	37 (25.2)	45 (23.7)			51 (21.8)		1.24 (0.71-2.17)	0.450	1.21 (0.72-2.06)	0.473	1.02 (0.57-1.84)	0.939	
Allele													
C	155 (52.7)	201 (52.9)			260 (55.6)		Reference		Reference		Reference		
A	139 (47.3)	179 (47.1)			208 (44.4)		1.12 (0.84-1.50)	0.445	1.11 (0.85-1.46)	0.439	1.00 (0.74-1.37)	0.964	
HWE	p=0.171	p=0.408			p=0.206								
VEGFA -2549 I/D (rs35569394)													
Genotype													
DD	39 (27.9)	52 (27.4)			78 (33.3)		Reference		Reference		Reference		
ID	67 (45.6)	93 (48.9)			110 (47)		1.22 (0.75-1.99)	0.430	1.27 (0.81-1.98)	0.297	0.96 (0.57-1.62)	0.880	
II	41 (27.9)	45 (23.7)			46 (19.7)		1.78 (1.00-3.15)	0.047	1.47 (0.85-2.52)	0.164	1.21 (0.67-2.20)	0.520	
Allele													
D	145 (49.3)	197 (51.8)			266 (56.8)		Reference		Reference		Reference		
I	149 (50.7)	183 (48.2)			202 (43.2)		1.35 (1.00-1.81)	0.043	1.22 (0.93-1.61)	0.146	1.11 (0.82-1.50)	0.516	
HWE	p= 0.285	p= 0.786			p= 0.521								
VEGFA -7 C/T (rs25648)													
Genotype													
CC	117 (79.6)	99 (52.1)			175 (74.7)		Reference		Reference		Reference		
CT	29 (19.7)	85 (44.7)			57 (24.4)		0.76 (0.46-1.26)	0.289	2.64 (1.74-4.00)	<0.001	0.29 (0.18-0.48)	<0.001	
TT	1 (0.7)	6 (3.2)			2 (0.9)		0.75 (0.07-8.34)	0.813	5.30 (1.05-26.78)	0.043	0.14 (0.02-1.19)	0.072	
Allele													
C	263 (89.5)	283 (74.5)			407 (87)		Reference		Reference		Reference		
T	31 (10.5)	97 (25.5)			61 (13)		0.79 (0.50-1.24)	0.305	2.29 (1.60-3.36)	<0.001	0.34 (0.22-0.53)	<0.001	
HWE	p=0.579	p=0.015			p=0.255								
MDM2 (rs3730485)													
Genotype													
CC	93 (63.2)			105 (55.3)	140 (59.8)		Reference		Reference		Reference		
CT	47 (32)			72 (37.9)	85 (36.3)		0.83 (0.53-1.3)	0.416	1.13 (0.75-1.69)	0.554	0.74 (0.46-1.17)	0.195	
TT	7 (4.8)			13 (6.8)	9 (3.9)		1.17 (0.42-3.25)	0.762	1.93 (0.79-4.67)	0.147	0.61 (0.23-1.59)	0.310	
Allele													
C	233 (79.3)			282 (74.2)	365 (78)		Reference		Reference		Reference		
T	61 (20.7)			98 (25.8)	103 (22)		0.93 (0.63-1.33)	0.680	1.23 (0.9-1.69)	0.198	0.75 (0.52-1.08)	0.127	
HWE	p=0.736			p=0.891	p=0.374								
Male Group													
	DR (n=267)	T2D (n= 235)			CN (n= 168)		DR vs. CN		T2D vs. CN		DR vs. T2D		
							OR (95% CI)	p-value	OR (95% CI)	p-value	OR (95% CI)	p-value	
VEGFA -2578 C/A (rs699947)													
Genotype													
CC	80 (30)	60 (25.5)			47(28)		Reference		Reference		Reference		
CA	134 (50.2)	124 (52.8)			78 (46.4)		1.00 (0.64-1.59)	0.968	1.25 (0.77-2.00)	0.366	0.81 (0.54-1.23)	0.320	
AA	53 (19.8)	51 (21.7)			43 (25.6)		0.72 (0.42-1.24)	0.241	0.93 (0.53-1.62)	0.796	0.78 (0.47-1.30)	0.338	
Allele													
C	294 (55.1)	244 (51.9)			172 (51.2)		Reference		Reference		Reference		
A	240 (44.9)	226 (48.1)			164 (48.8)		0.86 (0.65-1.13)	0.266	0.97 (0.73-1.29)	0.839	0.88 (0.69-1.13)	0.319	
HWE	p=0.818	p=0.383			p=0.358								
VEGFA -2549 D/I (rs35569394)													
Genotype													
DD	78 (29.9)	57 (24.3)			46 (27.4)		Reference		Reference		Reference		
ID	135 (50.6)	123 (52.3)			76 (45.2)		1.05 (0.66-1.66)	0.843	1.31 (0.81-2.12)	0.278	0.80 (0.53-1.22)	0.303	
II	54 (20.2)	55 (23.4)			46 (27.4)		0.69 (0.41-1.18)	0.179	0.96 (0.56-1.68)	0.899	0.72 (0.43-1.19)	0.200	
Allele													
D	291 (54.5)	237 (50.4)			168 (50)		Reference		Reference		Reference		
I	243 (45.5)	233 (49.6)			168 (50)		0.84 (0.64-1.10)	0.196	0.98 (0.74-1.30)	0.905	0.85 (0.66-1.06)	0.198	
HWE	p=0.750	p=0.472			p=0.217								
VEGFA -7 C/T (rs25648)													
Genotype													
CC	217 (81.2)	125 (53.2)			122 (72.6)		Reference		Reference		Reference		
CT	49 (18.4)	104 (44.2)			44 (26.2)		0.63 (0.39-1.00)	0.048	2.31 (1.50-3.55)	<0.001	0.27 (0.18-0.41)	<0.001	
TT	1 (0.4)	6 (2.6)			2 (1.2)		0.28 (0.03-3.13)	0.302	2.93 (0.58-14.79)	0.194	0.10 (0.01-0.81)	0.031	
Allele													
C	483 (90.4)	354 (75.3)			288 (85.7)		Reference		Reference		Reference		
T	51 (9.6)	116 (24.7)			48 (14.3)		0.63 (0.42-0.96)	0.033	2.0 (1.36-2.85)	<0.001	0.32 (0.23-0.46)	<0.001	
HWE	p=0.309	p=0.004			p=0.368								
MDM2 (rs3730485)													
Genotype													
CC	158 (59.2)			136 (57.9)	94 (56)		Reference		Reference		Reference		
CT	89 (33.3)			89 (37.9)	66 (39.3)		0.80 (0.53-1.21)	0.290	0.93 (0.62-1.41)	0.738	0.86 (0.59-1.25)	0.430	
TT	20 (7.5)			10 (4.2)	8 (4.7)		1.49 (0.63-3.51)	0.365	0.86 (0.33-2.27)	0.767	1.72 (0.78-3.80)	0.179	
Allele													
C	405 (75.8)			361 (76.8)	254 (75.6)		Reference		Reference		Reference		
T	129 (24.2)			109 (23.2)	82 (24.4)		0.99 (0.72-1.36)	0.934	0.94 (0.67-1.30)	0.690	1.05 (0.79-1.41)	0.720	
HWE	p=0.140			p=0.334	p=0.402								
NPDR and PDR groups													
	NPDR (n= 256)		PDR (n= 158)			T2D (n= 425)	NPDR vs. T2D		PDR vs. T2D		PDR vs. NPDR		
							OR (95% CI)	p-value	OR (95% CI)	p-value	OR (95%CI)	p-value	
VEGFA -2578 C/A (rs699947)													
Genotype													
CC	74 (28.9)		51 (32.3)			116 (27.3)	Reference		Reference		Reference		
CA	121 (47.3)		78 (49.4)			213 (50.1)	0.89 (0.62-1.29)	0.536	0.83 (0.55-1.27)	0.393	0.94 (0.59-1.48)	0.774	
AA	61 (23.8)		29 (18.4)			96 (22.6)	1.00 (0.65-1.54)	0.986	0.69 (0.40-1.17)	0.165	0.69 (0.39-1.22)	0.200	
Allele													
C	269 (52.5)		180 (57.0)			445 (52.4)	Reference		Reference		Reference		
A	243 (47.5)		136 (43.0)			405 (47.6)	0.99 (0.80-1.24)	0.947	0.83 (0.64-1.08)	0.161	0.84 (0.63-1.11)	0.215	
HWE	p=0.403		p=0.931			p=0.925							
VEGFA -2549 D/I (rs35569394)													
Genotype													
DD	66 (25.8)		51 (32.3)			109 (25.7)	Reference		Reference		Reference		
ID	126 (49.2)		76 (48.1)			186 (50.8)	1.12 (0.76-1.64)	0.563	0.87 (0.57-1.34)	0.533	0.78 (0.49-1.24)	0.295	
II	64 (25)		31 (19.6)			100 (23.5)	1.06 (0.68-1.64)	0.804	0.66 (0.39-1.12)	0.123	0.63 (0.36-1.10)	0.104	
Allele													
D	258 (50.4)		178 (56.3)			434 (51.1)	Reference		Reference		Reference		
I	254 (49.6)		138 (43.7)			416 (48.9)	1.03 (0.82-1.28)	0.811	0.81 (0.62-1.05)	0.110	0.79 (0.59-1.04)	0.097	
HWE	p=0.803		p=0.779			p=0.727							
VEGFA -7 C/T (rs25648)													
Genotype													
CC	205 (80.1)		129 (81.6)			224 (52.7)	Reference		Reference		Reference		
CT	50 (19.5)		28 (17.7)			189 (44.5)	0.29 (0.20-0.42)	<0.001	0.26 (0.16-0.40)	<0.001	0.89 (0.53-1.49)	0.656	
TT	1 (0.4)		1 (0.6)			12 (2.8)	0.09 (0.01-0.71)	0.022	0.14 (0.02-1.13)	0.065	1.59 (0.10-25.6)	0.744	
Allele													
C	460 (89.8)		286 (90.5)			637 (74.9)	Reference		Reference		Reference		
T	52 (10.2)		30 (9.5)			213 (25.1)	0.34 (0.24-0.47)	<0.001	0.31 (0.21-0.47)	<0.001	0.93 (0.58-1.49)	0.757	
HWE	p=0.261			p=0.695	p=0.001								
MDM2 (rs3730485)													
Genotype													
CC	153 (58.8)			98 (62)	241 (56.7)		Reference		Reference		Reference		
CT	86 (34.6)			50 (31.6)	161 (37.9)		0.88 (0.63-1.22)	0.447	0.76 (0.51-1.13)	0.181	0.87 (0.56-1.33)	0.515	
TT	17 (6.6)			10 (6.4)	23 (5.4)		1.16 (0.60-2.25)	0.651	1.06 (0.49-2.33)	0.866	0.92 (0.40-2.09)	0.839	
Allele													
C	396 (76.2)			246 (77.8)	643 (75.6)		Reference		Reference		Reference		
T	124 (23.8)			70 (22.2)	207 (24.4)		0.97 (0.75-1.26)	0.832	0.88 (0.65-1.20)	0.433	0.91 (0.65-1.27)	0.574	
HWE	p=0.449			p=0.299	p=0.337								

**Table 3:** Analyses of VEGFA and MDM2 polymorphisms using different genetic models in total samples, female group, and male group The data have been represented as OR: odds ratio and p-value. p-value < 0.05 was considered significant. Significant p-values are displayed in bold. CN: Healthy Controls, DR: Diabetic Retinopathy, HWE: Hardy-Weinberg Equilibrium, MDM2: Mouse Model Minute 2, T2D: Type 2 Diabetes, VEGFA: Vascular Endothelial Growth Factor

Total Samples							
		DR vs. CN		T2D vs. CN		DR vs. T2D	
Variant	Models	OR (95% CI)	p	OR (95% CI)	p	OR (95% CI)	p
VEGFA -2578 C/A (rs699947)	Dominant Model	1.03	0.840	1.19	0.261	0.87	0.354
	(CA + AA vs. CC)	(0.77-1.39)		(0.88-1.60)		(0.64-1.17)	
	Heterozygous Model	0.91	0.511	0.84	0.211	1.09	0.553
	(CC + AA vs.CA)	(0.69-1.20)		(0.64-1.10)		(0.83-1.42)	
	Recessive Model	0.91	0.574	0.96	0.786	0.95	0.767
	(AA vs. CC + CA)	(0.66-1.26)		(0.69-1.32)		(0.69-1.32)	
VEGFA -2549 I/D (rs35569394)	Dominant Model	1.13	0.419	1.29	0.097	0.88	0.394
	(ID + II vs. DD)	(0.84-1.53)		(0.95-1.75)		(0.65-1.19)	
	Heterozygous Model	0.90	0.471	0.83	0.190	1.08	0.556
	(DD + II vs.ID)	(0.69-1.19)		(0.63-1.09)		(0.83-1.42)	
	Recessive Model	1.00	0.983	1.04	0.827	0.97	0.842
	(II vs. DD + ID)	(0.72-1.39)		(0.75-1.43)		(0.70-1.33)	
VEGFA -7 C/T (rs25648)	Dominant Model	0.68	0.021	2.54	<0.001	0.27	<0.001
	(CT + TT vs. CC)	(0.49-0.94)		(1.89-3.40)		(0.20-0.36)	
	Heterozygous Model	1.45	0.031	0.42	<0.001	3.45	<0.001
	(CC + TT vs. CT)	(1.04-2.02)		(0.31-0.56)		(2.53-4.71)	
	Recessive Model	0.48	0.402	2.89	0.068	0.17	0.020
	(TT vs. CC + CT)	(0.09-2.65)		(0.92-9.04)		(0.04-0.75)	
MDM2 (rs3730485)	Dominant Model	0.91	0.482	1.06	0.662	0.85	0.249
	(ID + DD vs. II)	(0.68-1.20)		(0.81-1.40)		(0.65-1.12)	
	Heterozygous Model	1.23	0.159	0.99	0.924	1.25	0.128
	(II + DD vs. ID)	(0.92-1.64)		(0.74-1.31)		(0.94-1.66)	
	Recessive Model	1.58	0.150	1.30	0.429	1.25	0.448
	(DD vs. II + ID)	(0.85-2.95)		(0.68-2.46)		(0.70-2.22)	
Female Group							
		DR vs. CN		T2D vs. CN		DR vs. T2D	
Variant	Models	OR (95% CI)	p	OR (95% CI)	p	OR (95% CI)	p
VEGFA -2578 C/A (rs699947)	Dominant Model	1.11	0.641	1.17	0.449	0.95	0.821
	(CA + AA vs. CC)	(0.71-1.73)		(0.78-1.78)		(0.59-1.51)	
	Heterozygous Model	1.04	0.836	0.94	0.751	1.11	0.632
	(CC + AA vs.CA)	(0.69-158)		(0.64-1.38)		(0.72-1.71)	
	Recessive Model	1.21	0.447	1.11	0.644	1.08	0.753
	(AA vs. CC + CA)	(0.74-1.96)		(0.71-1.76)		(0.66-1.79)	
VEGFA -2549 I/D (rs35569394)	Dominant Model	1.38	0.162	1.33	0.186	1.04	0.864
	(ID + II vs. DD)	(0.88-2.18)		(0.87-2.02)		(0.64-1.70)	
	Heterozygous Model	1.06	0.785	0.93	0.691	1.14	0.539
	(DD + II vs.ID)	(0.70-1.60)		(0.63-1.36)		(0.74-1.76)	
	Recessive Model	1.58	0.064	1.27	0.316	1.25	0.380
	(II vs. DD + ID)	(0.97-2.56)		(0.80-2.02)		(0.76-2.04)	
VEGFA -7 C/T (rs25648)	Dominant Model	0.76	0.281	2.73	<0.001	0.28	<0.001
	(CT + TT vs. CC)	(0.46-1.25)		(1.81-4.11)		(0.17-0.46)	
	Heterozygous Model	1.31	0.293	0.40	<0.001	3.29	<0.001
	(CC + TT vs. CT)	(0.79-2.17)		(0.26-0.60)		(2.0-5.41)	
	Recessive Model	0.79	0.852	3.78	0.106	0.21	0.151
	(TT vs. CC + CT)	(0.07-8.84)		(0.75-18.96)		(0.03-1.76)	
MDM2 (rs3730485)	Dominant Model	0.86	0.503	1.21	0.344	0.72	0.139
	(ID + DD vs. II)	(0.57-1.32)		(0.82-1.78)		(0.46-1.11)	
	Heterozygous Model	1.21	0.385	0.93	0.739	1.30	0.260
	(II + DD vs. ID)	(0.78-1.88)		(0.63-1.39)		(0.82-2.04)	
	Recessive Model	1.25	0.665	1.84	0.172	0.68	0.425
	(DD vs. II + ID)	(0.46-3.43)		(0.77-4.39)		(0.26-1.75)	
Male Group							
		DR vs. CN		T2D vs. CN		DR vs. T2D	
Variant	Models	OR (95% CI)	p	OR (95% CI)	p	OR (95% CI)	p
VEGFA -2578 C/A (rs699947)	Dominant Model	0.91	0.657	1.13	0.584	0.80	0.270
	(CA + AA vs. CC)	(0.59-1.39)		(0.72-1.77)		(0.54-1.19)	
	Heterozygous Model	0.86	0.445	0.78	0.210	1.11	0.564
	(CC + AA vs.CA)	(0.58-1.27)		(0.52-1.15)		(0.78-1.57)	
	Recessive Model	0.72	0.160	0.81	0.363	0.89	0.610
	(AA vs. CC + CA)	(0.46-1.14)		(0.51-1.15)		(58-1.38)	
VEGFA -2549 I/D (rs35569394)	Dominant Model	1.66	0.029	1.38	0.167	1.21	0.389
	(ID + II vs. DD)	(1.06-2.63)		(0.87-2.18)		(0.79-1.84)	
	Heterozygous Model	0.81	0.280	0.75	0.160	1.07	0.691
	(DD + II vs.ID)	(0.55-1.19)		(0.51-1.12)		(0.76-1.53)	
	Recessive Model	1.09	0.680	0.85	0.478	1.29	0.212
	(II vs. DD + ID)	(0.71-1.68)		(0.54-1.33)		(0.87-1.92)	
VEGFA -7 C/T (rs25648)	Dominant Model	0.61	0.035	2.33	<0.001	0.26	<0.001
	(CT + TT vs. CC)	(0.39-0.97)		(1.53-3.57)		(0.18-0.39)	
	Heterozygous Model	1.93	0.005	0.45	<0.001	4.31	<0.001
	(CC + TT vs. CT)	(1.22-3.05)		(0.29-0.69)		(2.89-6.42)	
	Recessive Model	0.38	0.432	2.17	0.345	0.18	0.108
	(TT vs. CC + CT)	(0.03-4.23)		(0.43-10.91)		(0.02-1.47)	
MDM2 (rs3730485)	Dominant Model	0.88	0.507	0.92	0.701	0.95	0.767
	(ID + DD vs. II)	(0.59-1.29)		(0.62-1.38)		(0.66-1.35)	
	Heterozygous Model	1.29	0.207	1.06	0.774	1.22	0.289
	(II + DD vs. ID)	(0.87-1.93)		(0.71-1.59)		(0.85-1.76)	
	Recessive Model	1.62	0.263	0.89	0.808	1.82	0.132
	(DD vs. II + ID)	(0.70-3.77)		(0.34-2.30)		(0.83-3.98)	

For *VEGFA *-2578 C/A (rs699947) polymorphism, MAF was slightly lower in DR patients as compared to both T2D patients and CN in total samples and in the male group (Table 2). In the female group, MAF was slightly higher in DR patients compared to T2D patients and healthy controls (Table 2). No significant difference was observed with genotypes, alleles, and genetic models in any of the groups studied (Tables 2, 3).

MAF of *VEGFA *-2549 I/D (rs35569394) polymorphism was higher to some degree in DR cases than in T2D cases and CN in the female group (Table 2). In the female group, I allele and II genotype were significantly associated with an increased risk of DR compared to healthy controls (Table 2). Genetic model analysis revealed a significant increased risk of DR under the dominant model in the male group (Table 3). *VEGFA *-2549 I/D polymorphism was not associated with DR risk in total subjects (Table 2).

For *VEGFA *-7 C/T (rs25648) polymorphism, MAF was lower in DR cases compared to both T2D cases and CN in all groups (Table 2). The CT genotype was associated with a reduced risk of DR as compared to T2D cases and CN in total subjects, as well as in male and female groups (Table 2). Genetic model analysis showed a reduced risk of DR under the dominant model, whereas an increased risk of DR was observed under a heterozygous genetic model in all groups (Table 3). In total subjects, the recessive model showed protection towards DR cases vs T2D cases (Table 3). For rs25648 polymorphism, CT genotype and T allele were associated with reduced risk of PDR and NPDR as compared to T2D cases (Table 2).

MAF of *MDM2 *rs3730485 polymorphism was moderately lower in DR cases as compared to T2D cases and healthy controls in total subjects and in the female group (Table 2). In the male group, MAF was slightly higher in DR subjects compared to T2D subjects and healthy controls (Table 2). No significant difference was observed with genotypes, alleles, and genetic models in any of the groups studied (Tables 2, 3).

Linkage disequilibrium and haplotype analysis

Linkage disequilibrium (LD) analysis showed a strong LD between rs699947 and rs35569394 (D′ = 0.89, r2 = 0.78) (Figure 3). Haplotype analysis of rs69997-rs35569394-rs25648 polymorphisms showed that haplotype C-I-C and A-D-C were significantly associated with increased risk of DR as compared to T2D cases and CN, whereas haplotype A-I-T and C-D-T was significantly associated with reduced risk of DR as compared to T2D cases. Haplotypes A-I-T and C-D-T showed 2.29-fold and 1.96-fold risk for T2D as compared to CN (Table 4).

**Table 4:** Haplotype and genotype combinations data of VEGFA polymorphisms with DR risk Significant p-values are displayed in bold. p-value < 0.05 was considered significant. * VEGFA -2578C/A (rs699947) - VEGFA -2549I/D (rs35569394) - VEGFA -7C/T (rs25648). DR: Diabetic Retinopathy, T2D: Type 2 Diabetes, CN: Healthy Controls

Haplotype*	DR	T2D	CN	DR vs. CN		T2D vs. CN		DR vs. T2D	
	(%)	(%)	(%)	OR (95% CI)	p-value	OR (95% CI)	p-value	OR (95% CI)	p-value
C-D-C	46.0	43.6	48.4	Reference		Reference		Reference	
A-I-C	35.6	28.8	35.5	1.05 (0.84-1.31)	0.660	0.94 (0.74-1.18)	0.580	1.15 (0.90-1.46)	0.280
A-I-T	6.0	17.3	9.1	0.69 (0.45-1.05)	0.082	2.29 (1.62-3.24)	<0.001	0.30 (0.20-0.44)	<0.001
C-D-T	2.4	5.9	4.0	0.63 (0.30-1.32)	0.220	1.96 (1.05-3.66)	0.036	0.33 (0.16-0.65)	0.002
C-I-C	5.5	1.6	1.2	3.57 (1.77-7.19)	<0.001	1.03 (0.41-2.54)	0.960	2.95 (1.48-5.91)	0.002
A-D-C	3.0	1.0	1.4	1.97 (0.92-4.22)	0.080	0.71 (0.29-1.78)	0.470	3.54 (1.33-9.39)	0.012
Genotype* Combination									
	(n)	(n)	(n)						
CC-DD-CC	88	76	101	Reference		Reference		Reference	
CC-DD-CT	12	27	17	0.81 (0.37-1.79)	0.603	2.11 (1.07-4.15)	0.030	0.38 (0.18-0.81)	0.012
CC-ID-CC	17	4	2	9.76 (2.19-43.4)	0.003	2.66 (0.47-14.9)	0.266	3.67 (1.18-11.4)	0.024
CA-ID-CC	146	101	124	1.35 (0.93-1.96)	0.113	1.08 (0.73-1.61)	0.696	1.25 (0.84-1.86)	0.275
CA-ID-CT	27	98	51	0.61 (0.35-1.05)	0.074	2.55 (1.63-4.0)	<0.001	0.24 (0.14-0.4)	<0.001
CA-II-CC	11	2	2	6.31 (1.36-29.3)	0.019	1.33 (0.18-9.65)	0.779	4.75 (1.02-22.1)	0.047
AA-II-CC	50	36	58	0.99 (0.62-1.59)	0.965	0.82 (0.49-1.38)	0.461	1.2 (0.71-2.03)	0.499
AA-II-CT	23	45	27	0.98 (0.52-1.83)	0.944	2.21 (1.26-3.89)	0.006	0.44 (0.25-0.80)	0.007

Genotype-genotype interaction

The genotype combination data are given in Table 4. Genotype combinations of CC-DD-CT, CA-ID-CT, and AA-II-CT showed a significantly decreased risk of DR as compared to T2D cases. The power of the study was more than 80% for all the studied polymorphisms.

## Discussion

In this study, *VEGFA* (rs699947, rs35569394, and rs25648) and *MDM2* (rs3730485) polymorphisms were screened to determine the risk of DR in a group of T2D patients from Punjab, Northwest India. DR is a leading cause of visual loss in working-age populations [45,46]. For patients who survive for over 20 years with T2D, the majority suffer from DR [47,48]. The main progressors of DR are inflammation, angiogenesis, and apoptosis, leading to retinal cell death and eyesight loss [49,50]. VEGFA serves as the primary regulator in both normal and abnormal vascular development [51]. It has the potential to enhance retinal vascular permeability, destroy the blood-retinal barrier, and generate new blood vessels in DR, all of which are directly linked to the emergence and progression of DR [52]. The polymorphisms in promoter and 5ʹUTR lead to elevated transcriptional activity of *the VEGFA* gene, resulting in the increased production of VEGF as reflected in the serum VEGF levels of the individuals [53], as well as in vitro studies [54]. VEGF inhibition has been reported to cause nearly complete scaling down in retinal neovascularization, revealing the critical roles of VEGF in DR pathogenesis and management [55]. The p53 protein promotes apoptosis, and the VEGF protein promotes angiogenesis. *The MDM2* gene product is the down-regulator of p53 and has been reported as the upregulator of VEGF; both of these functions of *MDM2* increase the risk of DR [24]. There have been very few studies to test the association of rs699947, rs35569394, and rs25648 polymorphisms with DR risk worldwide, and no study was from Northwest India. There has been no previously reported study on the association of rs3730485 polymorphism with DR susceptibility worldwide. Most of the previous studies did not include healthy controls or diabetic controls (T2D individuals without DR) in their genetic analysis, which keeps us from more precise results. In the present study, the subject groups consisted of CN, T2D controls (without retinopathy), and individuals with DR (PDR and NPDR), for understanding the genetics of *VEGFA* -2578 C/A (rs699947), *VEGFA* -2549 I/D (rs35569394), *VEGFA *-7 C/T (rs25648), and *MDM2* (rs3730485) gene promoter region polymorphisms with the risk of DR in a population from Punjab, Northwest India. Details of the previous studies on DR with *VEGFA *rs699947, rs35569394, and rs25648 polymorphisms and their outcomes are given in Table 5.

**Table 5:** Summary of published studies of VEGFA rs69997, rs35569394, and rs25648 polymorphisms with DR DR: Diabetic Retinopathy, T2D: Type 2 Diabetes, CN: Healthy Controls

Variant	Country	Subjects			Results	Reference
		DR	T2D	CN		
rs699947	Iraq	103	31	36	No association	[51]
	Central India	105	51	-	No association	[13]
	Indonesia	33	35	-	Association of C allele with increased risk of DR	[56]
	Egypt	46	41	41	No association	[57]
	Egypt	74	74	-	No association	[58]
	Spain	14	26	-	Association of CA genotype with increased risk for DR	[19]
	China	129	139	-	Association of AA genotype with increased risk of DR	[59]
	Korea	253	134	260	Association of A allele with increased risk of DR	[60]
	Japan	177	292	-	Association of A allele and AA genotype with increased risk of PDR	[61]
	Finland	131	98	526	No association	[62]
	Australia	290	235	-	Association of AA genotype with increased risk of DR	[63]
	Japan	175	203	-	No association	[64]
rs35569394	Central India	105	51	-	No association	[13]
	Poland	38	62	-	Association of D allele with increased risk for DR	[65]
	Spain	14	26	-	Association of DD genotype with decreased risk and ID genotype with increased risk of DR	[19]
	Poland	195	92	493	Association of D allele with increased risk of DR	[40]
rs25648	South India	120	90	-	Association of T allele and CT genotype with increased risk of DR	[66]
	Japan	150	118	-	No association	[67]

*VEGFA* rs699947 polymorphism was significantly associated with DR in Korean [60], Japanese [61], Chinese [59], and Australian [63] populations, whereas no association with DR was observed in Iraq [51] and Egyptian [57,58] populations. A study from central India reported no association of rs699947 DR cases vs T2D cases [13]. A meta-analysis of nine studies showed that A allele and CA genotype were significantly associated with PDR risk in the overall Asian populations [68]. In the present study, there was no significant association of rs699947 with DR risk. Similarly, no association of rs699947 with DR has been reported in studies from Finland [62] and Japan [64].

The association of D-allele in rs35569394 with increased VEGF protein expression has been documented in the literature [69]. D allele was associated with an increased risk of DR in the Polish population [40,65]. The present study showed no significant difference in overall samples, but in the female group, II genotype and I allele showed a significantly increased risk of DR in comparison to CN. No association of rs35569394 with DR risk has been reported in a single previous study from central India [13].

There are very few studies on rs25648 in DR worldwide. In the present study on Northwest Indians, CT-genotype and T-allele showed significant protection to DR as compared to T2D cases and healthy controls. The model analysis showed significant protection in the dominant model and significant risk in the heterozygous model for DR cases vs. T2D cases. A study from Japan reported no significant association of rs25648 with DR [67]. CT-genotype and T-allele conferred significant risk towards DR in the South Indian population [66].

*MDM2* rs3730485 polymorphism is present in the promoter P1 region of *MDM2* and has been reported to control the expression of the gene [70]. A previous study has revealed a potential association between *MDM2* T309G and PDR. However, at present, there is a lack of direct evidence that supports the role of *MDM2* in DR [24]. No previous reported study has analyzed the association of *MDM2* rs3730485 polymorphism with DR. In the present study, there was no significant association of genetic variation with DR or T2D risk in the studied groups.

In the present study, a strong LD was observed between rs699947 and rs35569394. Similarly, complete LD between the C-allele of rs699947 and the D-allele of rs35569394 have been reported in Central Indian DR patients [13]. Haplotypes C-I-C and A-D-C conferred a risk of more than twofold in DR as compared to T2D cases, and haplotype C-I-C showed a significant risk of 3.57-fold towards DR as compared to CN in the present study (Table 4). Haplotypes A-I-T and C-D-T revealed significant protection to DR as compared to T2D. The C-D haplotype of rs699947 and rs35569394 polymorphisms was associated with enhanced VEGF expression [60]. Genotype-genotype combinations CC-ID-CC and CA-II-CC showed a significant risk of 3.67-fold and 4.75-fold in DR cases vs. T2D cases. The combinations of CC-DD-CT, CA-ID-CT, and AA-II-CT revealed significant protection in DR cases vs. T2D cases. The findings of the present study in Northwest Indians and previously reported studies in different populations [57,71] have different results, suggesting that ethnicity influences the association of VEGFA polymorphisms with DR.

Strengths and limitations of the study

Strengths: This is the first study to evaluate the association of four selected polymorphisms with DR in the studied population. DR has been studied in the context of its two types (PDR and NPDR), males and females, and their relation with the polymorphic variants. Additionally, this study includes the T2D patients as diabetic controls, which makes the results clearer.

Limitations: The present study was limited by not having expression analysis data of the *VEGFA *and *MDM2 *genes in the studied population. The selected genes were studied with only one and three polymorphisms, so more polymorphisms should be analyzed for better knowledge of the genetics of DR. Further, functional studies could be done. The study population could also be subdivided into ethnic groups for a better understanding of the genetics of DR. Additionally, the current study was carried out only on the population from the Punjab region, which could differ from the whole of Northwest India.

## Conclusions

In conclusion, the results of our study revealed that *VEGFA *-7 C/T (rs25648) polymorphism was significantly associated with a decreased risk of DR and that *VEGFA* -2549 I/D (rs35569394) polymorphism was significantly associated with an increased risk of DR in the female group. In the present study, haplotypes C-I-C and A-D-C were associated with more than twofold increased risk of DR. The present study was limited in not having corresponding evidence such as the level of gene and protein expression. This is the first study to investigate the association of *MDM2* rs3730485 polymorphism with DR and the first in recent years to study the association of *VEGFA* -7 C/T (rs25648) with DR. Analysis of *MDM2* rs3730485 and *VEGFA* -2578 C/A (rs699947) polymorphisms reported no significant association with the risk of DR in the studied population. The current study has furthered our knowledge about understanding the genetics of *VEGFA* and *MDM2* genes with DR in the Northwest Indian population, rooting for further future research on the topic.
